# Resveratrol and 2-Ethyl-6-Methyl-3-Hydroxypiridine N-Acetyl Cysteinate as Protecting Agents upon the Stress Exposure

**DOI:** 10.3390/ijms241713172

**Published:** 2023-08-24

**Authors:** Irina V. Zhigacheva, Irina F. Rusina, Natalia I. Krikunova, Aleksandr N. Goloschapov, Timur L. Veprintsev, Olga I. Yablonskaya, Aleksei V. Trofimov

**Affiliations:** 1 Emanuel Institute of Biochemical Physics, Russian Academy of Sciences, Ul. Kosygina 4, Moscow 119334, Russia; 2 N.N. Semenov Federal Research Center for Chemical Physics, Russian Academy of Sciences, Ul. Kosygina 4, Moscow 119334, Russia; 3 Moscow Institute of Physics and Technology (National Research University), Institutskii per. 9, Dolgoprudny 141701, Russia

**Keywords:** oxidative stress, ROS generation and scavenging, bioantioxidants, mitochondria, chemiluminescence assay, resveratrol, 2-ethyl-6-methyl-3-hydroxypiridine N-acetyl cysteinate

## Abstract

The increased generation of reactive oxygen species (ROS) by mitochondria under stress conditions leads to lipid peroxidation (LPO) as a consequence of the ROS interactions with polyunsaturated fatty acids in the lipid bilayer of cell membranes, causing their damage. It was assumed that chemical preparations that reduce the excessive ROS generation by mitochondria should exhibit protecting properties under oxidative-stress conditions. In this context, the antioxidants resveratrol (RSV) and 2-ethyl-6-methyl-3-hydroxypyridine N-acetylcysteinate (NAC-3-HP) were examined as potential chemical protectors upon the exposure to stress, able to maintain the functional state of mitochondria.

## 1. Introduction

The discovery of new protecting agents to maintain the pertinent functions of physiological systems in living organisms upon exposure to stress factors is of prime biomedical value. Clearly, when it comes to oxidative stress, bioantioxidants are considered as such potential protecting agents [1,2,3,4].

The primary mechanisms of developing the stress reaction of the body involve an increase in adrenaline and norepinephrine levels in the blood that lead to an increase in the intracellular content of Ca^2+^ ions and their accumulation in mitochondria [5]. The subsequent mitochondrial swelling is accompanied by an increased generation of reactive oxygen and nitrogen species (ROS and RNS, correspondingly) [6,7], causing oxidative modification of proteins and damage to mitochondrial and nuclear DNA [8]. In addition, the interaction of ROS with polyunsaturated fatty acids (FA), a part of the lipid fraction of mitochondrial membranes, leads to the activation of lipid peroxidation (LPO), resulting in the accumulation of toxic oxidation products, such as aldehydes and 4-hydroxy-2,3-nonenals. Such processes have a number of pathogenic consequences from inflammations to carcinogenic developments [8,9,10,11]. It can be assumed that substances and their compositions that reduce the excessive ROS generation by mitochondria, most prominently synthetic and natural antioxidants, should exhibit protecting properties towards oxidative stress.

**Figure 1:**
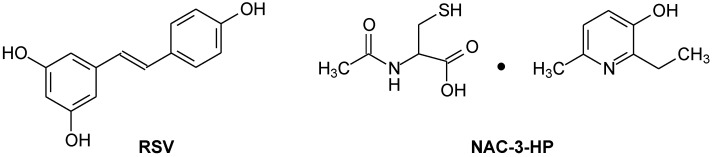
Chemical structures of resveratrol (RSV) and 2-ethyl-6-methyl-3-hydroxypyridine N-acetylcysteinate (NAC-3-HP).

In the present work, as potential protecting agents upon the exposure to stress, we examined two antioxidants: the natural antioxidant resveratrol (trans-3,4,5-trihydroxystilbene) (RSV) and a derivative of 3-hydroxypyridine, namely 2-ethyl-6-methyl-3-hydroxypyridine N-acetylcysteinate (NAC-3-HP) (Figure 1).

RSV is synthesized by plants in response to fungal damage or abiotic stress. At low (micromolar) concentrations, it activates a number of antioxidant enzymes due to the activation of the redox-sensitive transcription factors NF-kB/ARE [12,13]. Depending on its concentration and on the type of cells, RSV may also exhibit pro-oxidant properties, which lead to oxidative damage to cellular DNA in the presence of transition metals such as copper [12]. It should be mentioned that this pro-oxidant activity of RSV was associated with its antitumor and chemopreventive properties [14,15].

As for NAC-3-HP, it is noteworthy that the derivatives of 3-hydroxypyridine are heterocyclic analogs of aromatic phenols and, thus, also exhibit antioxidant and antiradical propensities [16]. Besides 2-ethyl-6-methyl-3-hydroxypyridine, NAC-3-HP contains acetylcysteine (NAC), which also exhibits antioxidant activity; the latter is accounted for by the ability of NAC to act as a precursor of reduced glutathione. Furthermore, upon depletion of endogenous cysteine and reduced glutathione, NAC acts as a direct antioxidant towards certain oxidants [17]. However, at high concentrations in the presence of transition metals, NAC exhibits oxidative activity. It may reduce transition metals and promote the ROS generation in Fenton-type processes or form thiyl radicals. Detailed knowledge of the influence on the functional state of mitochondria by NAC-3-HP is lacking to date. In the present work, we examine the effects of NAC-3-HP and RSV on mitochondria upon stressful developments.

## 2. Results

### 2.1. Antiradical Activity of RSV and NAC-3-HP

**Figure 2:**
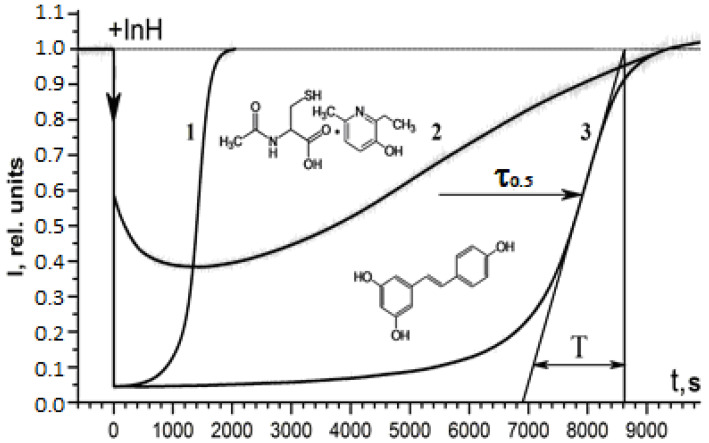
Time profiles of the chemiluminescence emission (intensity, I, in relative units) derived from the model reaction mixture of ethylbenzene (20% in chlorobenzene), which has been oxidized with the initiation rate *W_i_* = 9.2 × 10^−9^ Ms^−1^ at 50 °C in the presence of 2 × 10^−4^ M of DBA used as the chemiluminescence enhancer upon addition of 9.77 × 10^−6^ M of the standard chain-breaking antioxidant chromane C_1_, CrC_1_ (1), 9.80 × 10^−6^ M of NAC-3-HP (2) and 2.0 × 10^−6^ M of RSV (3).

The antiradical activity of RSV and NAC-3-HP was examined with the help of the chemiluminescence methodology [18,19,20,21,22] using a model chemiluminescent reaction of a hydrocarbon substrate (in the present work, ethylbenzene) oxidation initiated by azo-bis-isobutyronitrile (AIBN) at 50 °C, according to the experimental procedure whose details have been reported before [18,19,20,21,22] (as a relevant alternative to such a chemiluminescence methodology, the approach based on luminol chemiluminescence [23] should be mentioned). In oxidation of a hydrocarbon substrate (e.g., ethylbenzene), the excited-state generation followed by the light emission derives from the disproportionation of the two ethylbenzene peroxyl radicals RO_2_. Specifically, the two electronically excited products of such a step of the chain oxidation process are generated; the triplet ketone (in the present case, acetophenone) and singlet oxygen are both able to emit photons. However, it is customary to measure the light emission derived either from the excited ketone (direct phosphorescence) or from the pertinent emission-intensity enhancer, which is the appropriate luminophore that gets excited in the radiationless energy transfer from the triplet ketone. In the reported work, we used 9,10-dibromoanthracene (DBA) as the chemiluminescence enhancer. Introduction of the pertinent chain-breaking antioxidants, the inhibitors (InH) of the oxidation process (in this work, RSV and NAC-3-HP) that scavenge the RO_2_**.** radicals, results in the chemiluminescence quenching. Upon gradual consumption of the InH, the quasi stationary RO_2_**.** concentration restores, thereby recovering the chemiluminescence intensity level. Figure 2 exhibits the chemiluminescence time profiles upon introduction of the RSV and NAC-3-HP portions into the probe chemiluminescent “cocktail” of the hydrocarbon substrate (ethylbenzene), which has been oxidized. To control the initiation rate (*W_i_*) of the free-radical oxidation before and after measuring the chemiluminescence time profiles in the presence of RSV and NAC-3-HP, the standard antioxidant chromane C_1_, CrC_1_ (synthetic analog of α-tocopherol), was used. At a known initial concentration of chromane C_1_, [CrC_1_]_0_, the reaction initiation rate is determined with Equation (1) [18,19,20,21,22], in which τ_0_._5_ stays for the “dark” induction period (*cf*. Figure 2) of the chemiluminescence process [18,19,20,21,22], while *f* = 2 is the stoichiometric coefficient of CrC_1_, reflecting the number of peroxyl radicals (namely, 2) scavenged per molecule of this standard antioxidant.

*W_i_* = *f*[InH]_0_/τ_0.5_ = 2[CrC_1_]_0_/τ_0.5_ (1)

The kinetics of the recovery of the chemiluminescence intensity reflects the “strength” of the chain-breaking antioxidant expressed by the rate constant *k*_inh_ of the interaction of the InH with RO_2_**.** [18,19,20,21,22]. The *k*_inh_ value may be obtained from the maximal slope, (d*I*/dt)_max_ of the chemiluminescence intensity curve, *I*(t) (at the inflection point), according to Equation (2) [18,19], in which *k*_t_ is the rate constant of the RO_2_**.** disproportionation (for ethylbenzene *k*_t_ = 1.9 × 10^7^ M^−1^s^−1^), the termination step of the oxidation chain reaction [18,19].

(d*I*/d*t*)max = 1/*T* = 0.237(*k*_inh_/(2*k*_t_)^1/2^)*W_i_*^1/2^ (2)

**Figure 3:**
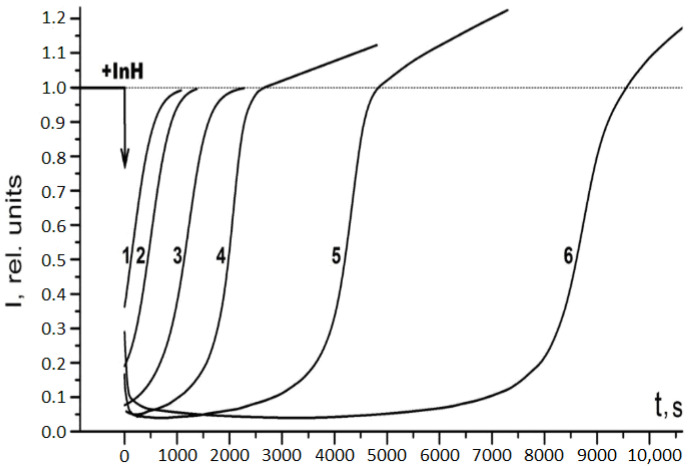
Time profiles of the chemiluminescence emission (intensity, I, in relative units) derived from the model reaction mixture of ethylbenzene (20% in chlorobenzene), which has been oxidized with the initiation rate *W_i_* = 9.2 × 10^−9^ Ms^−1^ at 50 °C upon the RSV addition in the following concentrations: 0.85 × 10^−6^ M (1), 1.67 × 10^−6^ M (2), 3.23 × 10^−6^ M (3), 10^−5^ M (4), 1.96 × 10^−5^ M (5) and 3.23 × 10^−5^ M (6) ([DBA] = 10^−5^ M).

Figure 3 exhibits the chemiluminescence kinetics at different RSV concentrations.

As is evident from Figure 3, at low RSV concentrations (RSV) ≤ 10^−6^ M after the consumption of the antioxidant, the chemiluminescence intensity returns to its initial level, while at (RSV) ≥ 10^−6^ M, the intensity exhibits an increased level (*ca.* 20%) after the induction period. The initiation rate (*W_i_*) measured with chromane C_1_ also becomes higher (up to 20%) at (RSV) ≥ 10^−6^ M, which is not the case for (RSV) ≤ 10^−6^ M. Clearly, these observations are indicative of the pro-oxidant effect of RSV.

The acquired *k*_inh_ and *f* data for RSV and NAC-3-HP as well as for acetylcysteine measured in the present work with the same methodology are collected in Table 1 along with the literature data on the *k*_inh_ and *f* values for other known chain-breaking antioxidants.

### 2.2. Antioxidant and Pro-Oxidant Activities of RSV and NAC-3-HP in Aging Mitochondria

The RSV and NAC-3-HP antioxidant activity was examined in the mitochondrial membranes using the mitochondria artificial-aging model (incubation in hypotonic salt medium) [24]. In all the experiments disclosed below, RSV and NAC-3-HP were dissolved in double-distilled water. Specifically, isolated mitochondria (2–3 mg of protein) were placed in 0.5 mL of medium containing 65 mM KCl, 10 mM HEPES and 1 mM KH_2_PO_4_ at pH 7.4. Their incubation proceeded 20–25 min at room temperature. The lipids were extracted with a mixture of chloroform:methanol (2:1 by volume) from mitochondria containing 3–5 mg of protein. The ratio of the mitochondria medium to the mixture of chloroform:methanol was 1:10.

**Figure 4:**
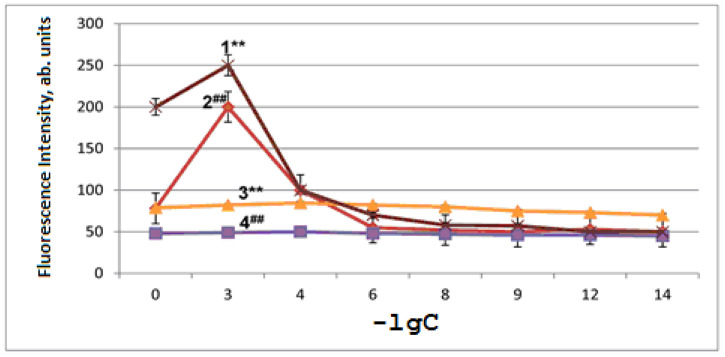
The effects of artificial aging of mitochondria and additions of RSV and NAC-3-HP (symbol C refers to the RSV and NAC-3-HP concentration in M) on the fluorescence intensity of the LPO products (per 1 mg of protein) in the membranes of mouse liver mitochondria: the RSV addition upon aging (1), the addition of NAC-3-HP upon aging (2), the RSV addition to control samples without aging (3) and the addition of NAC-3-HP to control samples without aging (4). Designations ** and ^##^ refer to *p* < 0.01.

The artificial mitochondria aging is associated with LPO activation, which was monitored by measuring the fluorescence of the Schiff bases that are formed between the protein amino groups and aldehydes (malonic dialdehyde and 4-hydroxy-2,3-nonenal). The end LPO products increased by 1.6 times (Figure 4). In Figure 4, curves 1 and 2 refer to mitochondria subjected to artificial aging upon the addition of RSV (curve 1) or NAC-3-HP (curve 2) to the incubation medium, while curves 3 and 4 pertain to control samples of mitochondria not subjected to aging upon the addition of RSV (curve 3) or NAC-3-HP (curve 4). In these experiments, 3 mL of chloroform and, subsequently, 0.3 mL of methanol were added to the cuvette. The fluorescence-excitation wavelength was 360 nm, while the emission intensity was recorded at 450 nm. The results of the measurements are expressed in arbitrary units of the fluorescence intensity recalculated per 1 mg of protein plotted versus the negative logarithm (−lgC) of the concentration (C) of RSV or NAC-3-HP, accordingly (Figure 4).

As is observed in Figure 4, the addition of RSV and NAC-3-HP to the incubation medium of mitochondria leads to the dose-dependent alteration of the fluorescence emission. Thus, while at high concentrations (10^−3^ to 10^−4^ M), both RSV and NAC-3-HP cause the increase in the LPO products’ fluorescence, thereby manifesting the pro-oxidant effect of both compounds in the mitochondrial membranes, at their low concentrations (<10^−5^ M), the antioxidant effect is evident through lowering of the fluorescence intensity.

### 2.3. LPO Activation in Mitochondrial Membranes under Conditions of Acute Hypobaric Hypoxia and Alcohol Poisoning

The protective properties of RSV and NAC-3-HP were investigated using models of acute hypobaric hypoxia (AHH) and acute alcohol poisoning (AAP), since under these conditions, free-radical oxidation processes are activated, thereby intensifying LPO [25,26]. Acute alcohol poisoning was caused by the oral administration of ethanol at the dose of 8 g/kg to the mice. The experimental group of mice was intraperitoneally injected with 2 × 10^−6^ mol/kg of RSV or 10^−6^ mol/kg of NAC-3-HP for 5 days before oral administration of ethanol. The control group of animals was divided into two subgroups. The first subgroup was injected with the same amounts of RSV or NAC-3-HP as the experimental animals. The injection was carried out at the same time as in the case of the experimental mice. The second subgroup was intraperitoneally injected with double-distilled water in portions whose volumes were equal to the volumes of the RSV or NAC-3-HP injections.

**Figure 5:**
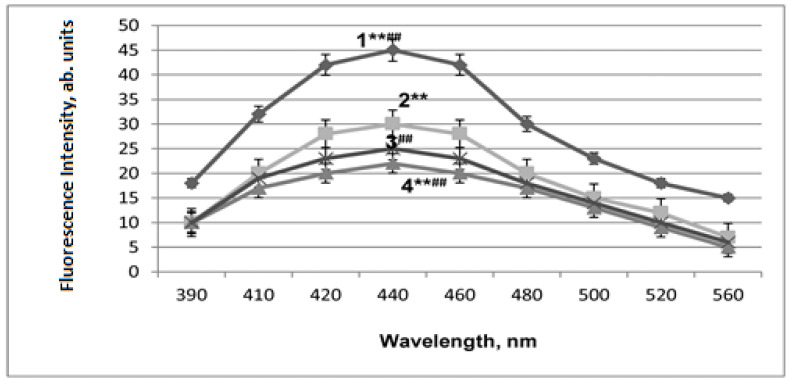
Fluorescence spectra of the LPO products (per 1 mg of protein) at excitation wavelength 360 nm: AAP (1), under the AAP conditions and upon administration of 2 × 10^−6^ mol/kg of RSV to the experimental animals during 5 days with the last injection 45 min before measurements (2), under the AAP conditions and upon administration of 10^−6^ mol/kg of NAC-3-HP to the experimental animals during 5 days with the last injection 45 min before measurements (3), and control samples, which refer to the animals not exposed to AAP and receiving either RSV or NAC-3-HP (4). Designations ** and ^##^ refer to *p* < 0.01.

As is evident from Figure 5, the administration of 2 × 10^−6^ mol/kg of RSV or 10^−6^ mol/kg of NAC-3-HP to the experimental animals for 5 days prevents LPO activation under the AAP conditions. The reason for using 2 × 10^−6^ mol/kg of RSV and 10^−6^ mol/kg of NAC-3-HP stems from the observation that 10^−6^ mol/kg of both antioxidants is efficient for LPO inhibition; however, in the case of RSV, the best results on the mice survival were obtained at 2 × 10^−6^ mol/kg. The magnitude of the fluorescence intensity of the LPO products in the mitochondrial membranes from the mice subjected to acute alcohol poisoning (AAP) and treated with RSV, NAC-3-HP is not shown in Figure 5 in view of nominal changes in the intensity level with such a treatment.

At the excitation wavelength of 360 nm, the fluorescence in the region at approximately 420 nm refers to the light emission derived from the 4-hydroxy-2,3-nonenal species, while the fluorescence at approximately 520 nm is attributed to that of malondialdehyde. In our experiments, these fluorescence bands overlap with the emission maximum at approximately 440 nm (Figure 5). Thus, one may conclude that 4-hydroxy-2,3-nonenal emission makes the major contribution to the fluorescence of the LPO end products.

Similar results were obtained under the AHH conditions.

### 2.4. Fatty Acid Composition of Mitochondrial Membranes under Conditions of Acute Hypobaric Hypoxia

**Figure 6:**
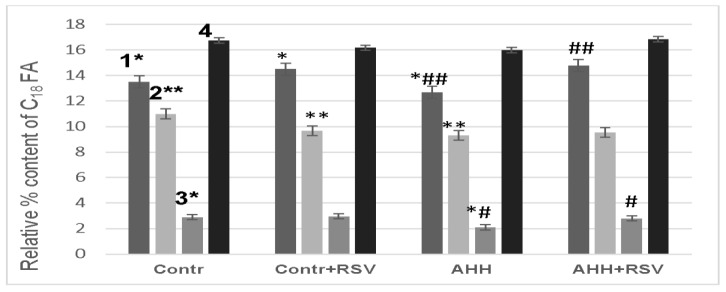
Influence of the AHH conditions and RSV on the relative percentage of C_18_ FA in the membranes of the mouse liver mitochondria for the four groups of animals: the control group (Contr), the control group upon the RSV administration (Contr + RSV), the group subjected to the AHH-induced stress (AHH) and the group subjected to the AHH-induced stress upon the RSV administration (AHH + RSV); the numbers 1–4 refer to: 18:2 ω6 (1), 18:1 ω9 (2), 18:1ω7 (3) and 18:0 (4). Designations * and ^#^ refer to *p* < 0.05, while ** and ^##^ pertain to *p* < 0.01.

**Figure 7:**
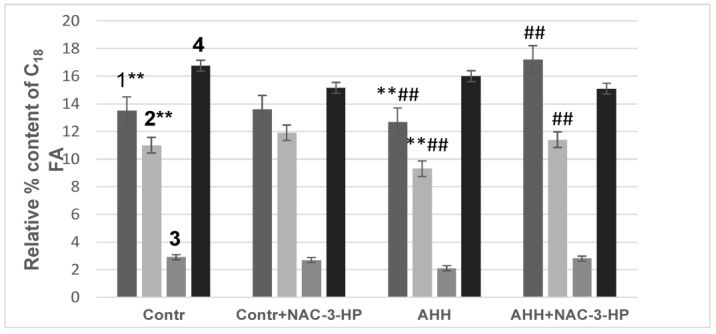
Influence of the AHH conditions and NAC-3-HP on the relative percentage of C_18_ FA in the membranes of the mouse liver mitochondria for the four groups of animals: control group (Contr), control group upon the NAC-3-HP administration (Contr + NAC-3-HP), group subjected to the AHH-induced stress (AHH) and group subjected to the AHH-induced stress upon the NAC-3-HP administration (AHH + NAC-3-HP); the numbers 1–4 refer to: 18:2 ω6 (1), 18:1 ω9 (2), 18:1ω7 (3) and 18:0 (4). Designations ** and ^##^ refer to *p* < 0.01.

Activation of LPO upon developing the oxidative stress should influence the fatty acids (FA) composition of the lipid fraction in the mitochondrial membranes. In the experiments to examine the effect of lipid peroxidation on the fatty acids composition of the lipid fraction of the mitochondrial membranes, the mice (one animal from each group) were slaughtered simultaneously and each at the same time (10 am). Indeed, we observed the changes in the content of fatty acids having 18 and 20 and 22 carbon atoms (FA having 18 carbon atoms: 18:2 ω6, 18:1 ω9, 18:1ω7, 18:0; FA having 20 carbon atoms: 20: 3ω6, 20: 4ω6, 20: 5ω3; FA having 22 carbon atoms: 22: 6ω3), which is exhibited in Figure 6 and Figure 7 (data in tabular form are given, accordingly, in Tables S1 and S2, Supplementary Materials).

Thus, the content of linoleic acid in the total lipid fraction of the mitochondrial membranes under the AHH conditions decreased by 6%, while the content of 18:1 ω9 decreased by 16%. Administration of RSV or NAC-3-HP to the experimental animals inhibited these changes. It should be noted that the RSV administration to the animals in the control group increased the content of 18:2 ω6 in the mitochondrial membranes by 7%.

Changes also occurred in the content of C_20_ FA: the pool 20:3 ω6 decreased by 18% under the AHH conditions, while the pool 20:5 ω3 lowered by 32%. In this case, C_20_ polyunsaturated FA/∑C_20_ monounsaturated FA decreased from 1.02 ± 0.08 to 0.77 ± 0.03. It is noteworthy that the administration of NAC-3-HP to the control group of animals leads to the decrease in the content of 20:4 ω6 and 22:6 ω3 in the total lipid fraction of the mitochondrial membranes by 5% and 3%, respectively. It is noteworthy that the injection of double-distilled water into the mice did not affect the fatty acid composition of the lipid fraction of the mitochondrial membranes.

### 2.5. Anti-Stress Properties of RSV and NAC-3-HP

Clearly, changes in the physicochemical properties of mitochondrial membranes should influence the functioning of these organelles and the pertinent metabolic processes and, thereby, may have the consequences for the resistance of the organism to stressful factors, which may be at least partially maintained with the help of the appropriate protecting agents. Indeed, administration of 2 × 10^−6^ mol/kg of the RSV to the experimental animals for 5 days increased their lifespan by 1.5–2.0 times and enhanced their survival rate by 10–16% under conditions of various types of hypoxia, while administration of 10^−6^ mol/kg NAC-3-HP to the animals for 5 days increased the lifespan by 1.6–1.9 times and the survival rate by 15–40% under conditions of cytotoxic and hemic hypoxia (Tables S3 and S4, Supplementary Materials). Under conditions of acute alcohol poisoning, it increased the lifespan by 3.8 times and survival rate by 12% (Table S4, Supplementary Materials).

## 3. Discussion

Examining the radical-scavenging properties of RSV and NAC-3-HP revealed the higher antiradical activity of the former compared to the latter compound. Nevertheless, in this context, NAC-3-HP exhibits antiradical activity similar to that of butylated hydroxytoluene (BHT, Dibunol) and Mexidol, well-known chain-breaking antioxidants and widely used drugs. RSV is a natural polyphenol bearing a stilbene-type structure, belonging to the phytoalexin family and exhibiting antioxidant activity in vivo [27]. However, it is noteworthy that such an antioxidant activity is not accounted for only by its radical-scavenging properties, and activation of the enzymatic antioxidant system should be taken into account [12]. In addition, RSV is capable of activating the nuclear transcription factor Nrf2, which regulates the expression of antioxidant genes [13]. At the same time, mitochondria are the major target for RSV. By inhibiting cAMP, RSV makes phosphodiesterase non-degradable, which leads to the increase in NAD^+^ levels and the activation of SIRT1. By increasing the expression of SIRT1, RSV reduces the production of mitochondrial ROS and increases the expression of Mn SOD [28].

As for NAC-3-HP, it constitutes a complex of an alkyl derivative of 3-hydroxypyridine and acetylcysteine. Like other 3-hydroxypyridines, it is a heterocyclic analog of aromatic phenols, containing hydroxyl and alkyl groups in the aromatic cycle, which account for a lipophilicity and the antiradical activity of such a compound. In addition, the presence of an alkyl chain in the aromatic cycle ensures that this compound readily enters living cells [29]. The 3-HP derivatives inhibit free radical oxidation in the lipid fraction of membranes, interact with lipid peroxide radicals and primary peptide radicals and increase the activity of SOD and other antioxidant enzymes, which makes them promising for protecting the body from oxidative stress [30]. Its inclusion in the complex with NAC ensures the solubility of the complex compound in protic solvents and increases its antioxidant activity. Its free thiol group is capable of interacting with electrophilic ROS groups [31]. The antioxidant activity of NAC can also be associated with its interaction with thiolated proteins, which leads to the release of free thiols as well as the restoration of proteins. The ability to restore disulfide bonds determines the ability of NAC to break mucopolysaccharide chains and depolymerize mucoproteins in sputum, which enables its use as a mucolytic and anti-inflammatory drug [17]. The recent attempts to use synthetic N-acetylcysteine (NAC) in the treatment of patients with the initial stages of COVID-19 are associated with its antioxidant properties, which help to improve alveolar gas exchange [32]. Possessing antiradical and antioxidant activity, RSV and NAC-3-HP prevent the oxidation of unsaturated C_18_ FA in the total lipid fraction of mitochondrial membranes, most prominently linoleic acid, one of the main FA that make up cardiolipin [33]. It should be noted that the RSV administration to the experimental animals of the control group increased the content of linoleic acid in the mitochondrial membranes. This is likely due to the induction of the expression of 5 and 6 desaturases (FADS1 and FADS2, respectively) [34], and it probably determines the protective effect of RSV. By preserving the pool of 18:2 ω6 in mitochondrial membranes under stress conditions, RSV probably contributes to the preservation of the cardiolipin pool, and this ensures the effective functioning of mitochondria and, consequently, the maintenance of the energy metabolism of cells, and contributes to the body’s resistance towards stress factors. Additional contribution to that may derive from the restoration of the pool of unsaturated C_20_ FA. Under the AHH conditions, the content of 20:3 ω6 and 20:5 ω3 decreased by two times. Considering that eicosanoids are signaling molecules and have a wide range of biological functions [35,36], the decrease in the content of these FA as well as the decrease in the content of linoleic acid should influence the body’s resistance to oxidative stress. Thus, RSV and NAC-3-HP, preventing the peroxidation of unsaturated FA containing 18 and 20 carbon atoms, may be considered as relevant protecting agents that increase the body’s resistance to stressful developments.

Concerning the manifestation of the pro-oxidant activity, particular attention should be paid to the qualitative agreement between the results of model chemiluminescence studies and studies on the antioxidants’ action in mitochondria (*cf*. Figure 3 and Figure 4). An increase in the concentration of antioxidants leads to a noticeable pro-oxidant effect, which must be taken into account when choosing optimal concentrations for their potential use as protective agents or adaptogens under developing oxidative stress.

## 4. Materials and Methods

### 4.1. Materials

Trans-3,4,5-trihydroxystilbene (resveratrol, RSV), Sigma-Aldrich, St. Louis, MO, USA, (HPLC grade, R5010, CAS number: 501-36-0) was used without further purification. 2-ethyl-6-methyl-3-hydroxypyridine N-acetylcysteinate (NAC-3-HP) was synthesized according to the known procedure [24] and generously provided by Dr. Yurii V. Kuzhetsov (Emanuel Institute of Biochemical Physics, RAS, Moscow, Russia). The other chemicals were obtained from standard suppliers and purified as published before [37].

### 4.2. Chemiluminescence Measurements

The antiradical activity of the studied compounds was assessed in a model reaction of ethylbenzene oxidation initiated by azo-bis-isobutyronitrile (AIBN) at 50 °C with the chemiluminescence methodology, whose details were published earlier [18,19,20,21,22]. Measurements of the chemiluminescence emission derived from the mentioned reaction mixture were carried out using the Hamamatsu photosensor module H7467 equipped with the RS-232C interface.

### 4.3. Fluorescence Measurements

The level of lipid peroxidation (LPO) was assessed with the fluorescence methodology [38] by measuring the fluorescence emission (excitation wavelength was 360 nm) derived from the Schiff bases formed between the protein amino groups and aldehydes (malonic dialdehyde and 4-hydroxy-2,3-nonenal), the end LPO products. Lipids were extracted with a chloroform–methanol mixture (2:1 by volume) from mitochondria containing 3–5 mg of protein. The fluorescence was measured with the Perkin Elmer LS 55 luminescence spectrometer.

### 4.4. Experiments with Animals and Biomaterials

As the experimental animals, male Balb/c 3-month-old mice (each group involved 20 animals) weighing 25–30 g each were used. RSV and NAC-3-HP were injected intraperitoneally for 5 days. For these experiments, RSV and NAC-3-HP were dissolved in double-distilled water and administered to the mice by 0.2 mL solution portions. RSV was administered to the mice at the level of 2 × 10^−6^ mol/kg, while NAC-3-HP was administered to the mice at the amount of 10^−6^ mol/kg.

The last administration of the drugs was carried out 45 min before the stress exposure.

The mice were on a standard diet.

In all the experiments, the preparations were administered not only to the mice subjected to stress but also to the control group of mice, which was not subjected to stress. In addition, the mice of the control group were administered with double-distilled water in volumes equal to the volume of the solutions of RSV and NAC-3-HP to examine the effect of the solvent. Expectedly, double-distilled water itself did not contribute to the observed effects of the RSV and NAC-3-HP preparations on the functional state of mitochondria.

The study was conducted under approval and supervision of the Institutional Ethics Committee of the Emanuel Institute of Biochemical Physics, Russian Academy of Sciences.

The isolation of mouse liver mitochondria was carried out with differential centrifugation [24]. The first centrifugation was performed at 600× *g* for 10 min, and the second was performed at 9000× *g* for 10 min. The pellet was re-suspended in the suspension medium containing 0.25 M sucrose and 10 mM HEPES at pH 7.4.

As before [24], for artificial aging of mitochondria, the isolated mitochondria (2–3 mg of protein) were placed in 0.5 mL of a medium containing 65 mM KCl, 10 mM HEPES and 1 mM KH_2_PO_4_ at pH 7.4 and were incubated for 20–25 min at room temperature.

The acquired experimental data are the mean values with their standard errors. The differences between the variants were considered significant at a value of *p* ≤ 0.05.

The study on the fatty acids (FA) composition of the liver mitochondrial membranes was carried out with gas–liquid chromatography and mass spectrometry.

Fatty acid methyl esters (FAME) were produced by acidic methanolysis of mitochondrial membrane lipids [38,39,40]. FAME were extracted with hexane, and the obtained solutions were analyzed. The FAME quantification was performed using a Kristall 2000 M chromatograph with a flame-ionization detector and quarts capillary column DB-1 (60 m × 0.32 mm, phase film thickness of 0.25 μm obtained from J&W Scientific). The FAME analysis was performed at a programmed temperature increase from 120 to 270 °C at the rate of 4 °C/min. The FAME content in the samples was calculated as the ratio of the peak area of a corresponding acid to the sum of the peak areas of all found FAME [41]. The standard deviation of the average values of the peak areas obtained in three measurements did not exceed 5% (relative). The identification of FAME in the samples was performed on the basis of mass spectra obtained after separation of the FAME under conditions similar to gas chromatographic analysis using a Hewlett Packard-6890 instrument. Mass spectra were obtained in the electron impact blow regime at an ionizing voltage of 70 eV and a scanning speed of 1C per decade of masses in the region of 40–400 Dalton.

The protective activity of the RSV and NAC-3-HP was studied in the Balb/c mice using models of acute hypobaric hypoxia and acute alcohol poisoning.

Modeling of the acute hypobaric hypoxia in the mice was carried out in the hyperbaric chamber at a low-pressure atmosphere (230.40 mm Hg), which corresponds to the height of 9000 m above sea level. During the first minute, the chamber created the rarefaction corresponding to 5000 m above sea level (this refers to the atmospheric pressure of 405 mm Hg). In each subsequent minute, “ascent” was carried out on the next 1000 m. The time of staying mice “at the height of 9000 m” above sea level was 5 min.

Acute alcohol poisoning was caused by the oral administration of ethanol at a dose of 8 g/kg to the mice weighing 25–28 g.

## Figures and Tables

**Table 1:** Characteristics of the antiradical activity (*k*_inh_ and *f* values) of chain-breaking antioxidants.

Antioxidant	10^4^ *k*_inh_ (Ms)^−1^	*f*
RSV ^1^	23.6 ± 0.04	2.1 ± 0.2
NAC-3-HP ^1^	3.84 ± 0.04	0.78 ± 0.2
Acetylcysteine ^1^	2.73 ± 0.04	0.32 ± 0.2
Butylated hydroxytoluene ^2^	2.0	1.9
Pyrocatechol ^3^	280	2.0
Mexidol (2-ethyl-6-methyl-3-hydroxypyridine) ^4^	4.7	1.9
CrC_1_ ^2^	452	2.0

^1^ Experimental conditions: *W_i_* = 5.0 × 10^−8^ (Ms)^−1^, 20% ethylbenzene in chlorobenzene, 50 °C. ^2^ From [19,21]. ^3^ From [20]. ^4^ From [21].

## Data Availability

Not applicable.

## Supplementary Materials

The following supporting information can be downloaded at: https://www.mdpi.com/article/10.3390/ijms241713172/s1.
