# Supplementary material for: Resveratrol and 2-Ethyl-6-Methyl-3-Hydroxypiridine N-Acetyl Cysteinate as Protecting Agents upon the Stress Exposure
Source: Int J Mol Sci. 2023 Aug 24;24(17):13172. doi: 10.3390/ijms241713172 (PMC10487494; doi:10.3390/ijms241713172)
Supplement: Supplementary file 1 [file ijms-24-13172-s001.zip › ijms-2512401-supplementary.pdf]

Supplementary Materials

Resveratrol and 2-Ethyl-6-Methyl-3-Hydroxypiridine N-Acetyl Cysteinate as Protecting Agents upon the Stress Exposure

Irina V. Zhigacheva [1], Irina F. Rusina [2], Natalia I. Krikunova [1], Aleksandr N. Goloschapov [1], Timur L. Veprintsev [1], Olga I. Yablonskaya [1] and Aleksei V. Trofimov [1,3,*]

[1] Emanuel Institute of Biochemical Physics, Russian Academy of Sciences; Ul. Kosygina 4, Moscow 119334, Russia; zhigacheva@mail.ru (I.V.Z.); kric@mail.ru (N.I.K.); golan@sky.chph.ras.ru (A.N.G.); timlev@yandex.ru (T.L.V.); olga.yablonsky@gmail.com (O.I.Y.)

[2] N.N. Semenov Federal Research Center for Chemical Physics, Russian Academy of Sciences; Ul. Kosygina 4, Moscow 119334, Russia; rusina939@mail.ru

[3] Moscow Institute of Physics and Technology (National Research University), Institutskii per. 9, Dolgoprudny 141701, Russia

* Correspondence: avt_2003@mail.ru; Tel.: +7-495-939-7358

Table S1. Influence of the AHH conditions and RSV on the relative percentage of C_{18} FA in the membranes of the mouse-liver mitochondria [1]

FA	Control [2]	Control and RSV [3]	AHH [4]	AHH and RSV [5]
18:2ω6	13.50 ± 0.15	14.00 ± 0.02	12.71 ± 0.10	14.35 ± 0.10
18:1ω9	11.00 ± 0.50	9.00 ± 0.12	9.50 ± 0.13	9.55 ± 0.95
18:1ω7	2.90 ± 0.04	2.80 ± 0.03	2.13 ± 0.30	2.60 ± 0.10
18:0	16.75 ± 1.30	16.00 ± 0.04	15.98 ± 1.00	16.85 ± 1.65
20:4ω6	20.30 ± 1.00	20.10 ± 0.50	20.93 ± 0.76	20.15 ± 1.35
20:3ω6	1.90 ± 0.02	1.80 ± 0.03	1.56 ± 0.03	2.05 ± 0.55
20:5ω3	0.50 ± 0.01	0.45 ± 0.03	0.20 ± 0.04	0.55 ± 0.15
20:0	9.60 ± 0.02	9.22 ± 0.01	13.00 ± 0.40	10.00 ± 0.05
22:6ω3	9.60 ± 0.01	10.00 ± 0.50	13.01 ± 2.04	10.00 ± 0.50
22:4ω6	0.55 ± 0.20	0.50 ± 0.15	0.40 ± 0.10	0.50 ± 0.01
22:5ω3	0.60 ± 0.01	0.56 ± 0.12	0.43 ± 0.11	0.60 ± 0.01

[1] Arbitrary units

[2] The control group (5-day injection of double-distilled water to mice in volumes corresponding to the injected volumes of RSV samples)

[3] The control group upon the RSV administration

[4] The group subjected to the AHH-induced stress

[5] The group subjected to the AHH-induced stress upon the RSV administration

Table S2. Influence of the AHH conditions and NAC-3-HP on the relative percentage of C_{18} FA in the membranes of the mouse-liver mitochondria [1]

FA	Control [2]	Control and NAC-3-HP [3]	AHH [4]	AHH and NAC-3-HP [5]
18:2ω6	13.50 ± 0.15	13.60 ± 0.03	12.70 ± 0.10	17.20 ± 0.04
18:1ω9	11.00 ± 0.50	11.90 ± 0.15	9.0 ± 0.13	11.40 ± 0.82
18:1ω7	2.90 ± 0.04	2.70 + 0.02	2.10 ± 0.40	2.80 ± 0.30
18:0	16.75 ± 1.30	15.00 + 0.05	15.98 ± 1.00	15.00 ± 0.65
20:4ω6	20.30 ± 0.01	19.25 ± 0.01	20.93 ± 0.76	20.26 ± 0.04
20:3ω6	9.60 ± 0.01	9.20 ± 0.01	7.85 ± 0.03	10.55 ± 0.02
20:5ω3	0.50 ± 0.01	0.52 ± 0.03	0.34 ± 0.02	0.58 ± 0.03
20:0	9.60 ± 0.02	8.05 ± 0.03	13.00 ± 0.40.	9.40 ± 0.02
22:6ω3	9.80 ± 0.05	9.40 ± 0.03	10.00 ± 0.40	9.64 ± 1.80
22:4ω6	0.40 ± 0.01	0.36 ± 0.01	0.23 ± 0.18	0.45 ± 0.30
22:5ω3	0.43 ± 0.11	0.40 ± 0.20	0.35 ± 0.05	0.41 ± 0.05

[1] Arbitrary units
[2] The control group (5-day injection of double-distilled water to mice in volumes corresponding to the injected volumes of NAC-3-HP samples)
[3] The control group upon the NAC-3-HP administration
[4] The group subjected to the AHH-induced stress
[5] The group subjected to the AHH-induced stress upon the NAC-3-HP administration

Table S3. Protective activity of RSV upon the stress exposure [1]

Impact	Measured parameters	Control [2]	RSV [3]
Modeling of ascent to a height of 11.5 thousand m (*hypobaric hypoxia)*	Lifetime (min)<br>% of survivors	4.0 ± 1.1<br>20 %	7.8 ± 1.1<br>30 %
Injection of sodium azide 20 mg/kg (*cytotoxic hypoxia*)	Lifetime (min)<br>% of survivors	3.2±0.8<br>0 %	7.4 ± 1.0<br>15 %
Injection of sodium nitrite 250 mg/kg (*hemic hypoxia*)	Lifetime (min)<br>% of survivors	15.1 ± 1.5<br>0 %	30.5 ± 320<br>16%

[1] 10 replicates per experiment

[2] The control group (mice exposed to various types of hypoxia and not receiving RSV)

[3] 2×10^{-6} mol/kg

Table S4. Protective activity of NAC-3-HP upon the stress exposure [1]

Impact	Measured parameters	Control [2]	NAC-3-HP [3]
Injection of sodium azide 20 mg/kg (*cytotoxic hypoxia*)	Lifetime (min)<br>% of survivors	3.3 ± 0.9<br>0%	5.2 ± 1.2<br>40%
Injection of sodium nitrite 250 mg/kg *(hemic hypoxia*)	Lifetime (min)<br>% of survivors	18.5 ± 2.1<br>0%	35.7 ± 4.4<br>15%
Ethanol injection 8 g / kg *(acute alcohol poisoning*)	Lifetime (min)<br>% of survivors	35.4 ± 6.1<br>0%	137.4 ± 31.1<br>12%

[1] 10 replicates per experiment

[2] The control group(mice exposed to cytotoxic or hemic hypoxia, or acute alcohol poisoning and not receiving NAC-3-HP)

[3] 10^{-6} mol/kg
